# Cost of Disorders of the Brain in Spain

**DOI:** 10.1371/journal.pone.0105471

**Published:** 2014-08-18

**Authors:** Oleguer Parés-Badell, Gabriela Barbaglia, Petra Jerinic, Anders Gustavsson, Luis Salvador-Carulla, Jordi Alonso

**Affiliations:** 1 IMIM - Health Services Research Group, Institut Hospital del Mar d’Investigacions Mèdiques, Barcelona, Spain; 2 CIBER en Epidemiología y Salud Pública (CIBERESP), Barcelona, Spain; 3 Pompeu Fabra University, Barcelona, Spain; 4 Quantify Research, Stockholm, Sweden; 5 Karolinska Institutet Alzheimer's Disease Research Center, Stockholm, Sweden; 6 Center for Disability Research and Policy, Faculty of Health Sciences, University of Sydney, Sydney, Australia; Hospital Nacional de Parapléjicos, Spain

## Abstract

**Background:**

Brain disorders represent a high burden in Europe and worldwide. The objective of this study was to provide specific estimates of the economic costs of brain disorders in Spain, based on published epidemiological and economic evidence.

**Methods:**

A cost-of-illness study with a societal perspective of 19 brain disorders was carried out. Cost data published between 2004 and 2012 was obtained from a systematic literature review. Direct healthcare, direct non-medical and indirect costs were considered, prioritizing bottom-up information. All costs were converted to Euro and to year 2010. The missing values were imputed with European estimates. Sensitivity analyses based on qualitative assessment of the literature and on a Monte Carlo simulation were performed.

**Results:**

The review identified 33 articles with information on costs for 11 disorders (8 neurological, 3 mental). The average per–patient cost ranged from 36,946 € for multiple sclerosis to 402 € for headache. The societal cost of the 19 brain disorders in Spain in 2010 was estimated in 84 € billion. Societal costs ranged from 15 € billion for dementia to 65 € million for eating disorders. Mental disorders societal cost were 46 € billions (55% of the total), while neurological disorder added up to 38 € billion. Healthcare costs represented 37% of the societal costs of brain disorders, whereas direct non-medical constituted 29% and indirect costs 33%.

**Conclusion:**

Brain disorders have a substantial economic impact in Spain (equivalent to almost 8% of the country's GDP). Economic data on several important brain disorders, specially mental disorders, is still sparse.

## Introduction

Brain disorders, that is, mental and neurological disorders, constitute 10.4% of the global burden of disease [1] and are projected to represent 14.4% in 2030 [2]. According to the World Health Organization, they accounted for 3.3% of the global deaths of individuals aged 15–49 years in 2010 [3]. In Europe, brain disorders contributed with about one quarter to the total burden of disease, a much greater proportion in comparison with other regions of the world [4]. Societal costs were estimated to be 798 € billion [5]. In Spain, in 2008, 1.5 million DALYs were lost due to brain disorders [6]. The vast majority of that burden is caused by the years lost due to disability (93.1% of the total DALYs) other than years of life lost [7].

Similarly to other Southern European countries, Spain has had high levels of social cohesion, and reliance on informal care, in contrast to some northern European countries. Such characteristics are associated with relatively low rates of people living alone and may cause lower rates of healthcare costs for residential care [9]. But family's ability to carry most of the burden may be limited due to several sociodemographic changes, such as smaller family size, increasing female participation in the labour market and higher divorce rates [10]. At the same time, societal expectations about a longer and more functional life expectancy are also increasing [8]. The economic cost of diseases is becoming an ever more important determinant for health policies and decision making [11] and thus solid Spanish specific estimates are needed. Cost-of-illness studies are of particular interest as they describe costs of every item related to disease, accordingly exposing the main factors that contribute to the societal costs of diseases.

### Aims of the study

Two previous systematic reviews concerning cost-of-illness of brain disorders in Europe included estimates for Spain: the EBC2005 [12] and the EBC2010 (Cost of disorders of the brain in Europe 2010) [5], [11]. In both cases the amount of literature found specifically containing cost information for Spain was scarce. We undertook a new Spanish-specific systematic review, with a broader dates span and the use of more databases, combined with the use of methods and data retrieved from the Cost of Disorders of the Brain in Europe 2010 study (EBC2010) [5].

The primary objective of our study was to provide the most updated and complete estimates of the economic costs of brain disorders in Spain, based on published epidemiological and economic evidence. Specific objectives were to estimate the societal cost per disorder and the per-patient cost; specifying direct and indirect costs in Spain for the year 2010.

## Methods

A systematic review of the literature was performed in order to obtain economic data inputs for a cost-of-illness study. A societal perspective and a bottom-up approach were used to estimate the cost of each of the 19 brain disorders (Table 1). Societal and per-patient costs were calculated taking into account three categories of costs. Finally, two sensitivity analysis were performed.

**Table 1:** Diagnostic groups included in the review and corresponding ICD-10 codes.

Mental disorders	ICD-10 codes	Neurological and neurosurgical disorders	ICD-10 codes
**Addiction**		Dementia	F00-F03
Alcohol dependence	F10.2	Epilepsy	G40
Cannabis dependence	F12.2	Headache	G44
Opioid dependence	F11.2	Multiple sclerosis	G35
**Anxiety disorders**		Neuromuscular disorders	G71, G61
Agoraphobia	F40.0		G12.21
Generalized anxiety disorder	F41.1	Parkinson's disease	G20
Obsessive-compulsive disorder	F42	Stroke	I61, I63
Panic disorder	F41.0		I64, I67
Post-traumatic stress disorder	F43.1		
Social phobia	F40.1		
Specific phobias	F40.2	Brain Tumor	C70–72
**Childhood and adolescence disorders**			D32–33
Conduct disorder	F91.x		D42–43
Hyperkinetic disorders/ADHD	F90.x	Brain traumatic injury	S06
Pervasive developmental disorders/autism	F84.x		
**Eating disorders**			
Anorexia nervosa	F50.0, F50.1		
Bulimia nervosa	F50.2, F50.3		
**Intellectual disability**	F70–F79		
**Mood disorders**			
Bipolar disorders	F30, F31		
Major depression	F32, F33		
**Personality disorders (PD)**			
Dissocial PD	F60.2		
Emotionally unstable PD	F60.3		
**Psychotic disorders**			
Schizophrenia and other psychotic	F2 x		
disorders and syndromes			
**Sleep disorders**			
Hypersomnia	G47.1		
Narcolepsy	G47.3		
Nonorganic insomnia	F51.x		
Sleep apnea	G47.4		
**Somatoform disorders**	F45		

### Cost data - Systematic review

A literature search was carried out using the databases PubMed (MEDLINE), ISI Web of Science and SCOPUS. A filter by publication date was applied. All papers published between 1^st^ of January 2004 and 1^st^ June 2012 were included. The search terminology included strings for dates, Spain [13] and each of the 19 brain disorders. The search was conducted on September 2012.

Studies were included if the following criteria was met: to incorporate descriptive information about cost or resource use; to report data of at least one type of cost; to study at least one of the brain disorders (Table 1); to include cost information form Spain; and to be written in English, Spanish or Catalan. Studies were excluded if economic data was available but could not extrapolated into a monetary form or could not be extrapolated to yearly per-patient costs. Articles reporting costs of patients in clinical trials were excluded.

Title, abstract and full text review was performed by two reviewers. Title review was performed applying a low-threshold review method. Abstract and full text review inconsistences between reviewers were solved by a third reviewer. Data was extracted by two reviewers and differences were solved by consensus. Data concerning methodology, costs and specific disease were extracted. Methodology information included, among others, the perspective, the time scope, the currency and the year of costing. All per-patient costs stated in the included articles were extracted, but classified according to the categorization used in EBC2010. Two authors were contacted and provided additional data on identified articles [14], [15].

A grey literature review was performed using the databases Google, Tripdatabase, Teseo and Tesis Doctorals en Xarxa. The main strings of the systematic search were used. An e-mail was sent to 24 investigators identified in the scientific literature review inquiring for unrevealed grey literature. Four of them answered our request for relevant documents.

### Epidemiological data

The number of patients with brain disorders in Spain was retrieved from the EBC2010 study epidemiological review, as reported elsewhere [4]. The prevalence data was stratified by age, gender and disease severity.

### Methodological approach: Cost-of-illness

This study follows the cost-of-illness methodology used in the EBC2010 enabling to assign a monetary value to a disease cost using the epidemiological and economic information available for Spain [16]. A societal perspective is presented, taking a comprehensive approach to estimating direct and indirect costs [17]. The bottom-up method (identifying patients with the disease and collecting their individual cost) was prioritized over top-down method for cost collection.

The per-patient cost is the average of the resource consumption of individual patients with a given disorder in a given time period. In this study, per-patient costs of each disorder, retrieved from the systematic review, were considered in three categories: (1) direct health care costs included inpatient care, outpatient care, drugs and medical procedures and devices costs; (2) direct non-medical costs comprised informal care, adaptation costs and transportation costs; and (3) indirect costs were restrained to permanent or temporal absence from work and early retirement. Indirect costs due to premature mortality, intangible costs and costs of crime were excluded because of lack of data or valuable methods. Indirect costs were valued using the human capital approach. Costs related to research were also excluded.

A year-prevalence approach was used to estimate the costs of the total number of cases of each disorder in the year 2010, in other words, the societal costs, that reflect the resource consumption of the overall population imputable to a disorder, in a given time period. As published elsewhere [5], the prevalence estimates where based in population aged 18 years and above in most disorders. For addictive disorders and anxiety disorders the age span went from 14 to 65 years and for child and adolescent disorders from 2 to 17 years. In the case of dementia only people over 65 years were considered.

### Data analysis

The consumer price index (CPI) for all-items [18] was used for adjustment by inflation when costs data obtained in the systematic review did not concern the year 2010. Estimates presented in currencies other than Euro were converted to Euro using nominal exchange rates from the European Central Bank [19].

Whenever cost information of a particular disorder was impossible to obtain from our literature review, the European median from EBC2010 [5] was imputed to our data. European medians had been adjusted for income, health care expenditure and wage level across countries.

The prevalence ratios were multiplied by the number of inhabitants in Spain in order to calculate the number of patients with each disorder. According to Eurostat, Spain had almost 46 million inhabitants in 2010 [20]. The number of patients with each disorder was multiplied by the specific estimates of the per-patient cost. Indirect costs were only applied to the working population (between 18 and 65 years) unless the indirect costs estimates were actually presented as an average of the total population of all ages.

Finally, our analysis included the calculation of the un-weighted mean of every cost whenever there were multiple studies for one disorder. The estimates of each type of costs were added up to obtain the per-patient cost of each disorder. Per-patient cost was multiplied by the number of patients with the disorder, for every one of the 19 disorders, to calculate the total societal cost. In addition, the distribution by types of cost was calculated by adding up all disorders direct healthcare costs, direct non-medical costs and indirect costs separately. This was performed for all disorders and stratified by mental and neurological disorders.

### Sensitivity analysis

Two sensitivity analyses were performed separately. First, a questionnaire [21] was used to assess the quality of the 33 papers included. Five items were evaluated: (1) Is the perspective of the analysis clearly specified? (2) Is the choice of study design properly justified? (3) Are all relevant costs and effects included? (4) Are they assessed and measured adequately? (5) Is uncertainty assessed using a sensitivity analysis or other techniques? Quality assessment was performed by two reviewers and solved by consensus. Sensitivity analysis was based on the replication of the analysis using only the articles that were considered to provide high quality information.

A second sensitivity analysis consisted in a Monte Carlo simulation [22] to control for the uncertainty generated by obtaining the cost data from different studies, allowing to make probabilistic estimates of the costs. Monte Carlo simulation is based in (1) the selection of the probabilistic distribution that best fit the variables and (2) a large number of random samples obtained from these distributions; yielding a statistical output. For our specific analysis we assumed triangular distributions that included the maximum, the minimum and the median observations of every type of cost (direct healthcare, direct non-medical and indirect costs) for the 7 disorders (dementia, epilepsy, headache, mood disorders, multiple sclerosis, and Parkinson's disease) that had more than one article included. For every disorder and cost, the minimum and the maximum were the lowest and the highest costs provided by any of the articles included in the review. The mean per-patient cost between the articles was fixed as the most probable value in the triangular distribution. One thousand iterations (random samples) were used to obtain the statistical outputs.

## Results

### Systematic review

**Figure 1:**
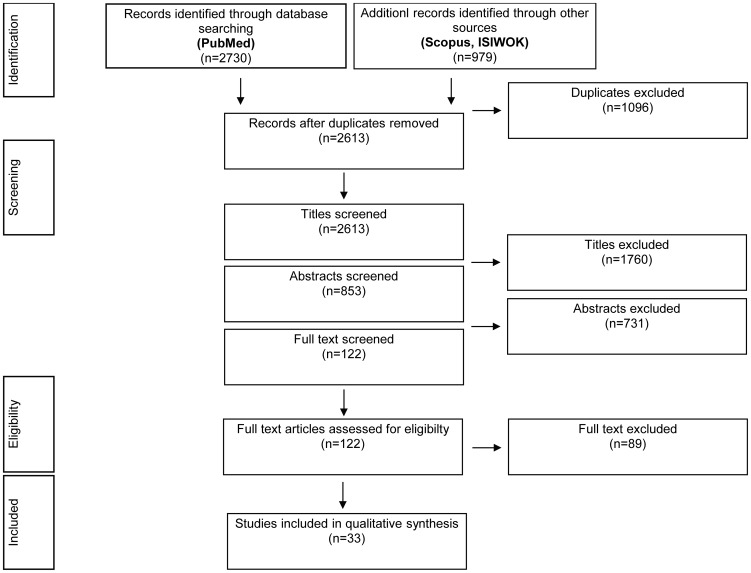
Flow of studies through the review process.

The search and screening process is summarised in Figure 1. The cost data literature review resulted in 33 relevant cost studies identified for 11 disorders (Table 2): anxiety disorder [23], dementia [24]–[31], epilepsy [32], [33], headache [15], [34], [35], mood disorders [36]–[40], multiple sclerosis [14], [41]–[43], Parkinson's disease [24], [44], psychotic disorders [45], [46], stroke [24], [47]–[50] and neuromuscular diseases [51]. Two articles were excluded from de model because they presented outlier estimations for stroke [52], [53] and traumatic brain injury [52].

**Table 2:** Summary of included articles and per-patient costs as stated in the article.

Article	Disorder group	Disorder	Year of costing	Currency	Direct health-care costs	Direct non-medical costs	Indirect costs	Total costs
Rovira, et al. (2012)	Anxiety disorders	Generalized anxiety disorder	2006	Euro	1,206	162	4,451	5.819
Coduras, et al. (2010)	Dementia	Alzheimer's disease	2006	Euro	4,272	12,708		16.980
Lopez-Bastida, et al. (2006)	Dementia	Alzheimer's disease	2001	Euro	3,289	24,281	628	28.198
Lopez-Pousa, et al. (2004)	Dementia	Alzheimer's disease	2001	Euro			6,649	6.649
Sicras, et al. (2005)	Dementia	Alzheimer's disease	2003	Euro	5,706	15,444		21.150
Turro-Garriga, et al. (2010)	Dementia	Alzheimer's disease	2006	Euro	8,212			8.212
Gustavsson, et al. (2011)	Dementia	Alzheimer's disease	2007	Pounds	4,189	18,504		22.693
Sicras, et al. (2005)	Dementia	Vascular dementia	2003	Euro	6,090	20,034		26.124
Oliva, et al. (2007)	Dementia	Alzheimer's disease	2002	Euro		11,110		11.110
Wimo, et al. (2007)	Dementia	Alzheimer's disease	2005	US dollar	6,219	14,989		21.208
Sancho, et al. (2008)	Epilepsy	Epilepsy	2005	Euro	4,982	255	1,618	6.855
Villanueva, et al. (2012)	Epilepsy	Epilepsy	2010	Euro	3,843	951		4.794
Badia, et al. (2004)	Headache	Migraine	2001	Euro	198		54	252
Bloudek, et al. (2012)	Headache	Migraine	2010	Euro	1,217			1.217
Linde, et al. (2012)	Headache	Migraine	2010	Euro	130		257	387
Linde, et al. (2012)	Headache	Tension type headache	2010	Euro	19		26	45
Linde, et al. (2012)	Headache	Medication overuse headache	2010	Euro	404		873	1.277
Linde, et al. (2012)	Headache	Other headaches	2010	Euro	22		0	22
Salvador-Carulla, et al. (2011)	Mood disorders	Unipolar/major depression	2006	Euro	4,002		1,346	5.348
Sicras-Mainar, et al. (2012)	Mood disorders	Unipolar/major depression	2009	Euro	620		1,275	1.895
Sicras-Mainar, et al. (2010)	Mood disorders	Unipolar/major depression	2006	Euro	1,579		1,810	3.389
Serna M, et al. (2007)	Mood disorders	Unipolar/major depression	2004	Euro	335		268	603
Gonzalez-Pinto, et al. (2010)	Mood disorders	Bipolar disorders	2003	Euro	283			
Kobelt, et al. (2006)	Multiple sclerosis	Multiple sclerosis	2005	Euro	12,142	12,540	8,145	32.827
Arroyo, et al. (2011)	Multiple sclerosis	Multiple sclerosis	2009	Euro	9,895	5,510		15.405
Casado, et al. (2006)	Multiple sclerosis	Multiple sclerosis	2004	Euro	7,775	21,297	16,618	45.690
Karampampa, et al. (2012)	Multiple sclerosis	Multiple sclerosis	2009	Euro	15,958	5,235	7,732	28.925
Cubo, et al. (2009)	Parkinson's disease	Parkinson's disease	2004	Euro	7,380	3,817	8,235	19.432
Oliva, et al. (2007)	Parkinson's disease	Parkinson's disease	2002	Euro		4,255		
Olivares, et al. (2008)	Psychotic disorders	Psychotic disorders	2005	Euro	5,569			
Vazquez-Polo, et al. (2005)	Psychotic disorders	Psychotic disorders	1999	Euro	3,989			
Beguiristain, et al. (2005)	Stroke	Stroke	2002	Euro	5,048			5.048
Hervas-Angulo, et al. (2006)	Stroke	Stroke	2004	Euro	2,271	1,863	426	4.560
Mar, et al. (2011)^b^	Stroke	Stroke	2008	Euro	16,341	10,932		27.272
Sicras, et al. (2008)	Stroke	Stroke	2006	Euro	1,591			1.591
Oliva, et al. (2007)	Stroke	Stroke	2002	Euro		4,478		4.478
Hervas, et al. (2007)^b^	Stroke	Stroke	2004	Euro		21,551		21.551
Navarrete-Navarro, et al. (2007)	Stroke	Stroke	2004	Euro	1,425	5,537	741	7.703
Hervas-Angulo, et al. (2006)^a^	Stroke	Stroke	2004	Euro	4,470	1,289	572	6.331
Navarrete-Navarro, et al. (2007)^a^	Stroke	Stroke	2004	Euro	5,173	6,420	2,347	13.940
Lopez-Bastida, et al. (2009)	Neuromuscular disorders	Amyotrophic lateral sclerosis	2004	Euro	8.018	19.602	8.575	36.195
Mar, et al. (2011)	Traumatic brain injury	Traumatic brain injury	2008	Euro	11.109	8.914		20.023

a Information provided by the article is incidence based, not prevalence based.

b outlier article excluded from the model.

A total of 2,936 grey literature documents were reviewed, but only two grey documents were found to be eligible for this review [54], [55]. In both cases information had also been published as scientific articles (that had already been identified in the scientific literature review), which were used for data extraction.

### Per-patient cost

**Figure 2:**
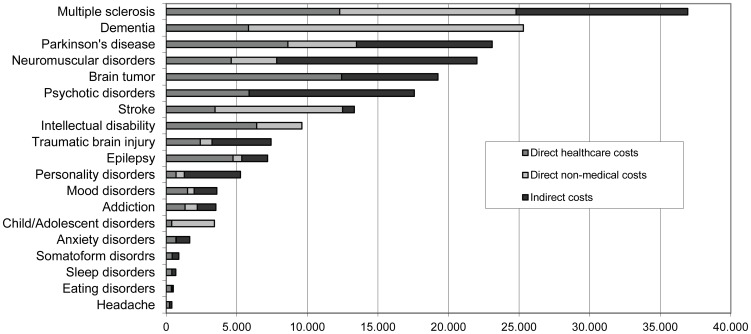
Per-patient cost by disorder and types of costs in Spain (€, 2010).

The estimated per-patient cost is displayed in Figure 2 (distribution) and Table 3 (numerals). The mean yearly per-patient cost was 2,440 €, although there was a wide variation depending on the diagnosis (ranging from 402 € for headache to 36,946 € for multiple sclerosis). The mean per-patient cost of mental disorders was higher (2,494 €) than that of neurological disorders (2,378 €). Nonetheless, when headache, a low-cost highly prevalent disorder, was not taken into account mean per-patient cost of neurological disorders was 16,309 €.

**Table 3:** Number of people, per-patient cost and societal cost by type of costs for all disorders in Spain 2010.

		Per-patient cost (€, 2010)		Societal costs (€ million, 2010)		
	Number of patients	Direct healthcare costs	Direct non-medical costs	Indirect costs	Total	Direct healthcare costs	Direct non-medical costs	Indirect costs	Total
Addiction^a^	1,437,560	1,335	867	1,315	**3,517**	1,919	1,247	1,890	**5,056**
Anxiety disorders	6,238,499	689	24	948	**1,661**	4,300	150	5,914	**10,365**
Brain tumor^a^	20,695	12,428	0	6,826	**19,254**	257	0	141	**398**
Child/Adolescent disorders^a^	480,074	392	3,021	0	**3,413**	188	1,450	0	**1,638**
Dementia	608,711	5,830	19,473	0	**25,303**	3,549	11,853	0	**15,402**
Eating disorders^a^	131,049	364	45	90	**499**	48	6	12	**65**
Epilepsy	225,346	4,734	619	1,827	**7,180**	1,067	140	412	**1,618**
Headache	13,909,125	233	0	168	**402**	3,244	0	2,341	**5,585**
Intellectual disability^a^	376,777	6,409	3,203	0	**9,612**	2,415	1,207	0	**3,622**
Mood disorders^c^	3,002,725	1,514	469	1,601	**3,584**	4,546	1,410	4,807	**10,763**
Multiple sclerosis	36,193	12,291	12,495	12,160	**36,946**	445	452	440	**1,337**
Neuromuscular disorders^d^	23,003	4,605	3,227	14,185	**22,016**	106	74	326	**506**
Parkinson's disease	79,789	8,614	4,866	9,612	**23,091**	687	388	767	**1,842**
Personality disorders^a^	396,532	697	583	3,979	**5,259**	276	231	1,578	**2,085**
Psychotic disorders^b^	453,650	5,870	0	11,705	**17,576**	2,663	0	5,310	**7,973**
Sleep disorders^a^	4,072,265	396	0	284	**680**	1,611	0	1,158	**2,769**
Somatoform disorder^a^	1,852,405	426	0	465	**891**	789	0	861	**1,650**
Stroke	644,025	3,461	9,032	835	**13,329**	2,229	5,817	538	**8,584**
Traumatic brain injury^a^	335,260	2,412	830	4,183	**7,426**	809	278	1,403	**2,489**
**Total**	**34,323,684**	**908**	**720**	**813**	**2,440**	**31,149**	**24,703**	**27,897**	**83,749**

a European imputation was used for all costs.

b European imputation was used for indirect costs.

c European imputation was used for unipolar depression direct non-medical costs and bipolar disorder indirect and direct non-medical costs.

d European imputation was used for neuromuscular disorders except for amyotrophic lateral sclerosis.

### Societal costs

**Figure 3:**
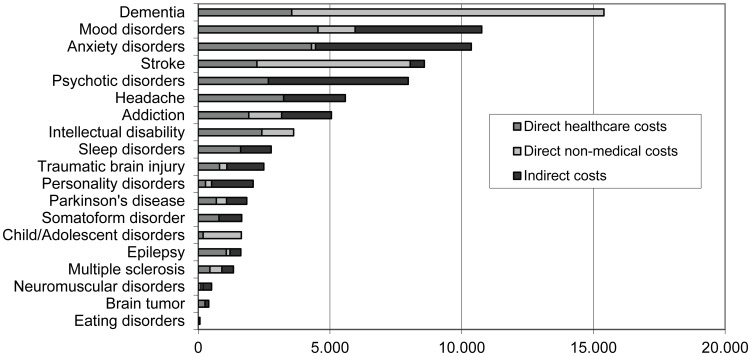
Societal cost by disorder and types of costs in Spain (€ million, 2010).

The societal cost of brain disorders in Spain 2010 was estimated at 84 € billion. Based on a total number of citizens in Spain of almost 46 million, the average cost of brain disorders per inhabitant per year in Spain was 1,725 €. Societal costs by disorder and cost type are shown in Figure 3 (distribution) and Table 3 (numerals). The most costly brain disorder in Spain was dementia with 15,402 € million.

### Distribution of the type of costs

Mental disorders accounted for 46 € billion, representing 55% of the societal costs of all brain disorders considered. Societal costs of neurological disorders added up to 38 € billion (45% of the total). Dementia was the most costly disorder, accounting for almost 20% of the societal costs.

**Figure 4:**
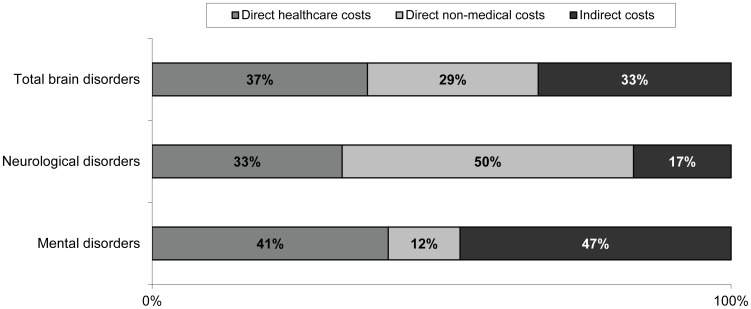
Distribution of types of costs in brain disorders, neurological disorders and mental disorders.

Overall, the majority of the estimated costs of brain disorders (Figure 4) were direct healthcare costs (37%) while direct non-medical costs constituted 29% and indirect costs 33%. Within neurological disorders direct non-medical costs constituted 50% of the costs, indicating a high dependence on informal care. On the contrary, mental disorders were driven mainly by indirect costs (47%), followed by direct healthcare costs (41%).

### Sensitivity analyses

#### Quality assessment

Among the articles included in the review, 15 were deemed to be high quality articles. For psychotic disorders no high quality information was found, whereas for epilepsy and neuromuscular disorders all articles included were considered high quality articles. For five disorders costs were recalculated using only the high quality articles stated: dementia [24], [30], [31], headache [15], [35], mood disorders [36], multiple sclerosis [14], [41], [42], Parkinson's disease [24] and stroke [47], [48], [50].

Costs were recalculated using the same methods but only high quality articles for six disorders, of which five resulted in higher estimates. Societal costs estimations increased in 509 € million for dementia; 6,827 € million for mood disorders; 109 € million for multiple sclerosis 33 € million for Parkinson's disease and 245 € million for stroke. A diminish of the estimation was only observed in headache, with a decrease of 1,562 € million. Taking all disorders into account, societal costs for brain disorders were estimated to be 86 € million, an increase of 6,161 € million, an additional 8%. In terms of per-patient cost, the increase was an average of 179 € per patient.

#### Monte Carlo simulation

The Monte Carlo simulation outputs were meant to show the inherent uncertainty of our estimations, driven by the use of different articles as our data source on cost information. Following, with a probability of 90%, the distribution of the per-patient costs for each disorder is shown: dementia 31,787 € (90% CI: 25,697 – 39,119); epilepsy 7,180 € (90% CI: 6,517 – 7,821); headache 432 € (90% CI: 317 – 566); mood disorders 4,238 € (90% CI: 2,763 – 5,999); multiple sclerosis 39,929 € (90% CI: 32,401 – 48,814); Parkinson's disease 23,091 € (90% CI: 22,810 – 23,371) and stroke 17,072 € (90% CI: 9,547 – 25,948). When the 7 disorders were analysed together, the Monte Carlo simulation showed that with a probability of 90% the median per-patient cost ranged from 1,696 € to 4,392 €. The probability of a median per-patient cost above 3,000 € was 36.5%. In comparison with the mean observed costs, the ones obtained through simulation were a 14.8% higher.

## Conclusion

### Main study findings

The present study shows that the economic burden of brain disorders in Spain was almost 84 € billion, mental disorders accounted for 46€ billon and neurological disorders for 38€ billion. This total figure corresponds to nearly 8% of the gross domestic product of Spain and surpasses the public healthcare expenditure of Spain, which was about 64 € billion in 2010 [54]. Brain disorders had a societal cost of about ten times higher than that of cardiovascular diseases (estimated at 7 € billion in Spain in 2003 [55]) or diabetes (close to 8 € billion in 2009 [56]). Although caution is needed due to differences in costing methodologies, brain disorders exceeded the economical weight of two of the most burdensome diseases in Spain.

### Strengths and weaknesses of the study

Our estimates were obtained through a previously tested method that permits the use of all evidence even if the articles included in the review used different methodologies or if only partial information was available. Our method offers comparable estimates across brain disorders and types of costs [5].

On the other hand, a number of limitations must be taken into account. First, double counting is likely to have occurred. This is reflected on the total number of individuals with one brain disorder, which was a sum of the number of patients for every disorder but did not take into account that some of these disorders coexist in the same individual. While we did not consider overlap between any pair of disorders, the original articles did evaluate the excess cost of a given disorder to the extent possible, linking expenditures to a singular disorder and taking into account the additional cost that a person with the disorder causes, irrespective of whether they have any other disorders or not. Second, the costs reported by the articles included in this review are dependent on the sampling of patients. Samples should be representative of the general population in the proportion of mild and severe cases. In this review, costs were obtained by the un-weighted mean of the costs stated in the original articles, but our data included a relatively small number of studies for some diagnostic categories with, sometimes, small number of patients. Thus, the relative distribution of mild and severe cases in each disorder may be exaggerating cost differences across disorders. Third, we did not find any information about the costs of 9 disorders, so we had to impute the European median values [5]. Such values may not correspond to the real costs for Spain, although it is unclear whether they would be higher or lower. Fourth, there are no international guidelines for cost-of-illness analyses, which hampers the use of quality standards for the assessment of available literature and the international comparison in contrast with other areas of health economics such as cost-effectiveness analysis.

Conversely, we are certain that our results systematically underestimate the costs of brain disorders, based on a number of considerations. One is that there were no estimates for Spain neither from any European country on direct non-medical costs for brain tumors, headaches, psychotic disorders, sleep disorders and somatoform disorders, and on indirect costs for intellectual disability. These concepts could not be included in the final estimates, resulting in an underestimation of the total costs. Also, indirect costs for disorders in children and adolescents as well as for dementia were assumed to be null because we presumed affected people were not part of the working population. We also underestimated the societal costs of brain disorders since intangible, crime related and mortality costs were not assessed. And also because some less prevalent (but sometimes more costly) disorders [5] could not be included in our study due to lack of data. In conclusion, our results provide a conservative estimate of the costs of brain disorders in Spain, as supported by the results of our sensitivity analyses.

### Comparison with previous studies

Our societal costs estimates of 83,749 € million due to brain disorders in Spain are consistent but an 8% higher than the estimates for our country provided by the EBC2010 estimates for Spain [5]. Higher estimates in our study are attributable to the inclusion of more recent articles in the review that take into account the increase of medical, pharmacological and informal care costs over the years. Specifically, our review identified 20 relevant studies for Spain that were not taken into account in EBC2010. Also of notice is the fact that European and Spanish costs distribution diverge. In Spain direct non-medical costs were proportionally more important than in Europe, while indirect costs tended to be lower. A previous study [57] estimated the socioeconomic costs of mental illness in Spain to total 7,018 € million in 2002. Our estimation was sixfold higher, 45,986 € million. That study used a top-down approach using administrative data from only the Canary Islands not disaggregated by disorder. It is likely that methodological differences on the types of costs and number of disorders considered explain results differences.

Specific estimates for other European countries have been published using the same methodology of ours [58]–[60] and results differ somewhat. The most (dementia) and least (headache) costly disorders are concordant across countries, but the societal costs vary. For instance, the mean per-patient cost of brain disorders in Spain was 2,440 € while in Switzerland it was 2,624 €. This difference is consistent with the higher health expenditure of Switzerland.

### Policy implications

Despite limitations, the results of this study should be useful for policymakers. One of the “grand challenges” [61] for brain disorders is to reduce the cost of effective medication and to provide effective and affordable community-based care and rehabilitation. To compare future policies for the reduction of costs associated with mental and neurological disorders with the present policies will only be possible if economic evaluation studies have been endorsed. While cost-of-illness studies have been criticized for not permitting to establish whether a country is spending too much or too little on a disease, they can help to inform decisions concerning allocation of funding. They can do so by providing a measure of the economic burden of particular health problems [55]. They can neither predict if higher resource allocation entails higher health earnings, but they enable a global vision of what is currently being spent, and what kind of costs are more relevant, being a useful tool for financial planning. Several regional health authorities in Spain have recently shown interest in including costs-of-illness studies to support planning of their health budgets [62]. In addition, the Spanish Ministry of Health has included within its disease management plans estimations of the cost-of-illness for diabetes mellitus, ischemic heart disease and cancer [57]. It has also funded cost studies of neurological diseases but not of mental diseases. These studies have not even been considered in the national mental health strategy which may explain the unbalance of available information between mental and neurological disorders in Spain [63].

### Further research

The vast economic burden of brain disorders and the policy implications emphasize the need of increased efforts in research. Gaps on knowledge on cost-of-illness of mental disorders and some neurological disorders should be addressed in the near future, when costs are likely to increase due to the aging population and the higher prevalence of degenerative disorders and associated disability. Moreover, as many other national health systems, the Spanish healthcare system is under restructuration through a new legislation [64], changes in organization and structures [65] and the effects of the economic crisis. Cost-of-illness studies on brain disorders with different societal, healthcare system and patient perspectives, as well as their distribution within the Spanish society should be accomplished for a better evaluation of policy changes.
